# Upregulation of Id1 by Epstein-Barr Virus-encoded LMP1 confers resistance to TGFβ-mediated growth inhibition

**DOI:** 10.1186/1476-4598-9-155

**Published:** 2010-06-18

**Authors:** Angela KF Lo, Christopher W Dawson, Kwok W Lo, Yanxing Yu, Lawrence S Young

**Affiliations:** 1 Cancer Research UK Cancer Centre, School of Cancer Sciences, University of Birmingham, Vincent Drive, Edgbaston, Birmingham, B15 2TT, UK; 2 Department of Anatomical and Cellular Pathology, Li Ka Shing Institute of Health Science, The Chinese University of Hong Kong, Prince of Wales Hospital, Shatin, Hong Kong; 3 Haematopoiesis and Immunology Program, Sidney Kimmel Cancer Center, Johns Hopkins School of Medicine, Baltimore, Maryland 21231, USA

## Abstract

**Background:**

Epstein-Barr virus (EBV)-encoded LMP1 protein is commonly expressed in nasopharyngeal carcinoma (NPC). LMP1 is a prime candidate for driving tumourigenesis given its ability to activate multiple signalling pathways and to alter the expression and activity of variety of downstream targets. Resistance to TGFβ-mediated cytostasis is one of the growth transforming effects of LMP1. Of the downstream targets manipulated by LMP1, the induction of Id1 and inactivation of Foxo3a appear particularly relevant to LMP1-mediated effects. Id1, a HLH protein is implicated in cell transformation and plays a role in cell proliferation, whilst Foxo3a, a transcription factor controls cell integrity and homeostasis by regulating apoptosis. The mechanism(s) by which LMP1 induces these effects have not been fully characterised.

**Results:**

In this study, we demonstrate that the ability of LMP1 to induce the phosphorylation and inactivation of Foxo3a is linked to the upregulation of Id1. Furthermore, we show that the induction of Id1 is essential for the transforming function of LMP1 as over-expression of Id1 increases cell proliferation, attenuates TGFβ-SMAD-mediated transcription and renders cells refractory to TGFβ-mediated cytostasis. Id1 silencing in LMP1-expressing epithelial cells abolishes the inhibitory effect of LMP1 on TGFβ-mediated cell growth arrest and reduces the ability of LMP1 to attenuate SMAD transcriptional activity. In response to TGFβ stimulation, LMP1 does not abolish SMAD phosphorylation but inhibits p21 protein expression. In addition, we found the induction of Id1 in LMP1-expressing cells upon stimulation by TGFβ. We provide evidence that LMP1 suppresses the transcriptional repressor ATF3, possibly leading to the TGFβ-induced Id1 upregulation.

**Conclusion:**

The current data provide novel information regarding the mechanisms by which LMP1 suppresses TGFβ-induced cytostasis, highlighting the importance of Id1 in LMP1 mediated cell transformation

## Background

The Epstein-Barr virus (EBV)-encoded latent membrane protein (LMP1) is commonly expressed in nasopharyngeal carcinoma (NPC) and is believed to play important role in NPC pathogenesis [1]. LMP1 is an oncogenic protein, inducing lymphomagenesis in transgenic mice and transforming rodent fibroblasts *in vitro*, rendering them tumourigenic *in vivo*. *In vitro *studies show that LMP1 is essential for EBV immortalisation of primary B cells, and can induce a state of cell activation in B lymphoma-derived cell lines. In epithelial cells, LMP1 increases cell proliferation, promotes anchorage independent growth, protects cells from apoptosis, induces an epithelial-mesenchymal transformation, promotes cell invasion and perturbs epithelial cell differentiation [2,3]. LMP1 is an integral membrane protein comprising a 24 amino acid N-terminal cytoplasmic domain, six transmembrane spanning domains connected by short reverse turns, and a 200 amino acid C-terminal cytoplasmic domain. LMP1 functions as a constitutively active viral mimic of CD40, engaging multiple signalling pathways which include NFκB, PI3K/Akt, ERK-MAPK/JNK, JAK/STAT, and p38/MAPK pathways to alter various gene expression programs [2,3].

Of the signalling pathways activated by LMP1, PI3K/Akt, ERK-MAPK and NFκB signalling pathways have been shown to induce phosphorylation and inhibit the activity of the Forkhead box class O (Foxo) transcription factors [4]. Foxo family members including Foxo1, Foxo3a, Foxo4 and Foxo6 activate or repress genes such as Bim, p27^kip ^and cyclin D1, which regulate apoptosis or cell-cycle progression respectively. Foxo proteins are subject to regulation through phosphorylation, resulting in nuclear to cytosolic export and subsequent degradation. Foxo protein deregulation is associated with cell proliferation, altered differentiation and an accumulation of DNA damage findings suggestive of a role in driving carcinogenesis [4,5]. Although a number of Foxo targets have been identified, a recent study in leukemic cells has shown that Foxo3a negatively regulates the transcription of Inhibitor of DNA binding 1 (Id1), a member of the helix-loop-helix (HLH) proteins [6]. The Id1 protein is unable to bind DNA, but it functions as dominant negative regulator, inhibiting the binding of other basic HLH (bHLH) transcription factors to their target genes. Over-expression of Id1 has been observed in a variety of cancers where it may contribute to a variety of cellular functions that include cell proliferation, resistance to apoptosis, angiogenesis, invasion and inhibition of terminal cell differentiation [7].

Cell proliferation and differentiation are tightly regulated by growth promoting factors and growth inhibitory factors. TGFβ functions as a prototypical tumour suppressor, inhibiting the growth of untransformed epithelial, endothelial and lymphoid cells. In keeping with its role as a tumour suppressor, resistance to TGFβ is regarded as one of the crucial steps in malignant progression [8,9]. TGFβ-mediated cell inhibition is induced by SMAD-dependent regulation of TGFβ target genes. LMP1-expressing fibroblasts and EBV-infected lymphocytes are reportedly refractory to TGFβ-mediated growth arrest [10,11]. Although several reports have demonstrated a role for NF-κB in modulating the transcriptional activity of SMAD complexes, the mechanism(s) by which LMP1 confers resistance to TGFβ are not fully resolved [12,13].

In this study, we report that LMP1 inactivates the function of Foxo3a leading to upregulation of Id1. The induction of Id1 by LMP1 confers cellular resistance to TGFβ through a mechanism involving inhibition of TGFβ-SMAD-mediated transcription. In addition, we show that LMP1 inhibits the expression of ATF3, a transcription repressor that co-operates with SMAD to mediate Id1 suppression. By inhibiting ATF3 expression, LMP1 relieves the suppressive effect of TGFβ on Id1 expression.

## Results

### LMP1 suppresses the expression and transcriptional activity of Foxo3a

**Figure 1:**
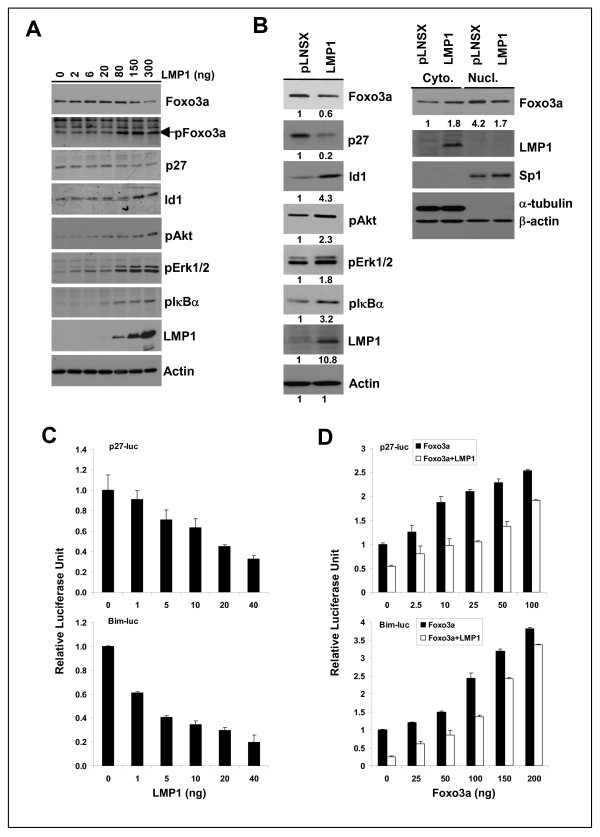
**LMP1 suppresses the expression and transcriptional activity of Foxo3a**. (A) HEK-293 cells were transfected with increasing amount of the LMP1 vector (pSG5-LMP1) as indicated. Forty-eight hours post-transfection, cells were harvested for immunoblotting analysis. For the detection of Id1 and Foxo3a, cells were cultured in serum free medium for 6 hrs before harvesting. (B) Total cell lysates of NP69-pLNSX control and NP69-LMP1 cells were analyzed by immunoblotting. For detection of cytoplasmic and nuclear proteins, cells were treated with protease inhibitor, MG132 (20μM) for 4 hrs prior to harvesting. Relative protein expression was calculated using densitometry with the control set at 1. (C) HEK-293 cells were transfected with various doses of the LMP1 expression vector (pSG5-LMP1) and reporter constructs for p27^kip ^or Bim. (D) HEK-293 cells were transfected with various doses of GFP-Foxo3a and the p27^kip ^or Bim promoter reporter constructs, together with 40 ng LMP1 expression vector (pSG5-LMP1) or control empty vector (pSG5). Cells were harvested for luciferase reporter analysis 48 hrs post-transfection. Luciferase activity was normalized to β-gal activity. Data shown are the mean ± s.d. of three separate experiments. The relative luciferase unit (RLU) is plotted relative to that of the reporter alone (set at 1).

LMP1 confers growth and transforming properties to epithelial cells by activating multiple signal cascades. These include the PI3K/Akt, ERK-MAPK and NFκB signalling pathways amongst others. Activation of these three pathways results in suppression of the transcriptional activity of Foxo3a [4]. One consequence of Foxo3a inactivation by LMP1 is inhibition of DNA repair [14]. Here, we examine additional downstream consequences of Foxo3a inactivation by LMP1. In keeping with previously published findings, we demonstrate that transient expression of LMP1 in HEK-293 cells stimulated Akt, Erk1/2 and IκB phosphorylation in a dose dependent manner and was accompanied by Foxo3a phosphorylation and protein degradation (Figure 1A) [2,14]. In agreement with previously published studies, reduction of p27^kip^, a transcriptional target of Foxo3a by LMP1 was also observed [15]. An examination of a nasopharyngeal epithelial cell line NP69 stably expressing LMP1 (NP69-LMP1) revealed increased cytoplasmic levels of Foxo3a, and an overall reduction in total Foxo3a and p27^kip ^protein (Figure 1B). Stable expression of LMP1 was also accompanied by increased phosphorylation of IκB, Akt and Erk1/2. To examine further the effect of LMP1 on Foxo3a-mediated transcription, luciferase assays were performed using promoter reporter constructs of two established Foxo3a target genes: p27^kip ^and Bim. As shown in Figure 1C, transient expression of LMP1 in HEK293 cells attenuated the activity of both promoter reporters in a dose-dependent manner. In the reciprocal experiment, exogenous expression of Foxo3a enhanced the activities of both p27^kip ^and Bim promoter reporters. This induction was antagonised by LMP1 expression (Figure 1D). Taken together, these data confirm that LMP1-induced phosphorylation, nuclear translocation and degradation of Foxo3a ablate Foxo3a transcriptional activity in epithelial cells.

### Inactivation of Foxo3a by LMP1 stimulates Id1 expression

**Figure 2:**
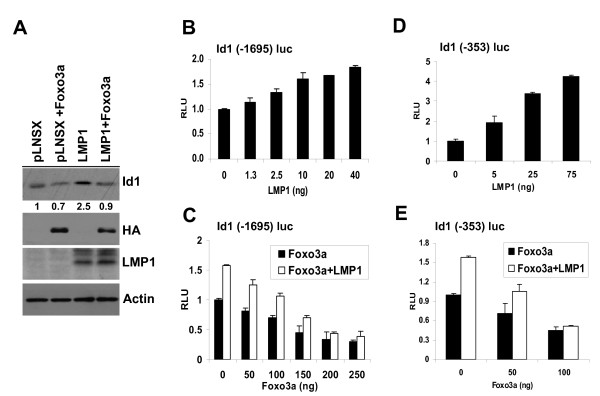
**Inactivation of Foxo3a by LMP1 results in Id1 induction**. (A) NP69 cells stably expressing pLNSX vector control or LMP1-pLNSX were transfected with a HA-tagged Foxo3a expression vector. Forty-eight hours after transfection, cells were cultured in growth factor free medium for 6 hrs prior to immunoblotting analysis. (B) HEK-293 cells were transfected with an Id1 (-1695) promoter luciferase reporter and various doses of LMP1 expression vector (pSG5-LMP1) or (C) various doses of Foxo3a expression vector together with 40 ng LMP1 expression vector or control pSG5 empty vector. (D) NP69 cells were transfected with an Id1 (-353) promoter luciferase reporter and various doses of a LMP1 expression vector (pSG5-LMP1) or (E) various doses of a Foxo3a expression vector together with 40 ng LMP1 expression vector or pSG5 control vector. Thirty hours post-transfection, cells were incubated in serum free medium for 6 hrs prior to harvesting for luciferase analysis.

A recent report has shown that Foxo3a downregulates Id1 transcription [6]. This led us to investigate whether inactivation of Foxo3a by LMP1 impacted on Id1 expression. Firstly, immunoblotting confirmed increased levels of Id1 expression in epithelial cells expressing LMP1 (Figure 1A &1B), findings that are consistent with previously published data [16,17]. Furthermore, transient overexpression of HA-tagged Foxo3a in NP69-LMP1 cells resulted in a marked reduction in Id1 protein expression (Figure 2A), confirming the reciprocal relationship between Foxo3a and Id1 expression. Using an Id1 (-1695) promoter reporter construct, we found that transient expression of LMP1 augmented Id1 promoter activity in HEK-293 cells in a dose-dependent manner (Figure 2B). While Foxo3a inhibited the transcriptional activity of Id1 promoter, LMP1 counteracted this negative effect (Figure 2C). Foxo3a has been shown to repress Id1 transcription through direct binding to the Id1 promoter at position -134 to -128 bp upstream of the ATG [6]. To evaluate further the interplay between Foxo3a, Id1 and LMP1, a shorter Id1 promoter construct (-353) was transfected into NP69 nasopharyngeal epithelial cells together with increasing amounts of LMP1. As shown in Figure 2D, LMP1 increased the luciferase activity of this shorter Id1 promoter construct. In addition, the suppressive effect of Foxo3a on this shorter Id1 promoter element was antagonised by LMP1 (Figure 2E). Taken together, these data confirm that LMP1 limits the ability of Foxo3a to repress Id1 promoter transcription.

### LMP1 induction of Id1 confers resistance to TGFβ-mediated cytostasis

**Figure 3:**
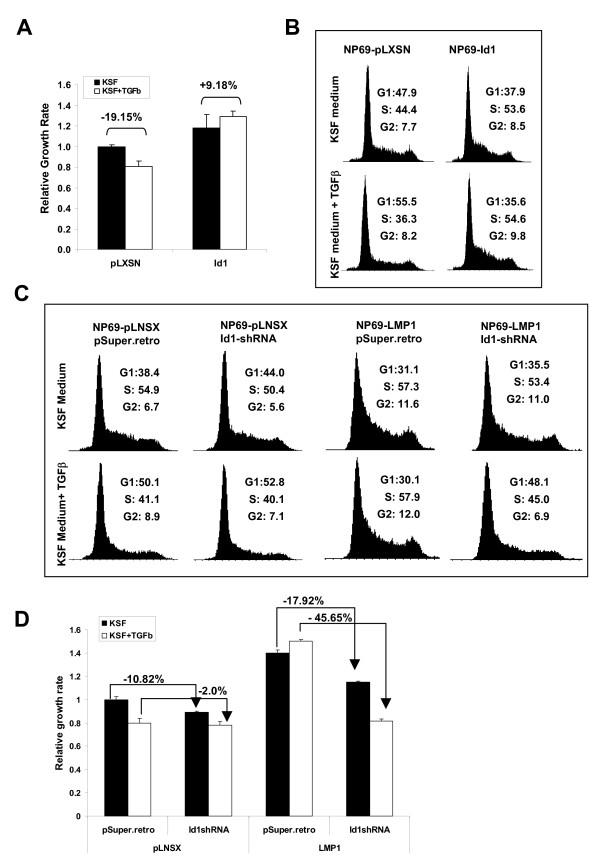
**LMP1 induction of Id1 confers resistance to TGFβ-mediated growth inhibition**. (A) NP69 cells stably expressing Id1 were treated with 10 ng TGFβ in KSF medium (KSF) for 24 hrs prior to cell growth analysis by MMT assay (A) and cell cycle analysis by flow cytometry (B). NP69-pLNSX and NP69-LMP1 cells expressing pSuper.retro control or Id1 shRNA (shId1B+C) were treated with TGFβ for 24 hrs prior to cycle analysis (C) and cell growth analysis (D). Data shown are the mean ± s.d. from three experiments.

TGFβ is a potent regulator of squamous epithelial homeostasis acting as a tumour suppressor by inducing cell cycle arrest. Id1 has multiple oncogenic functions imparting resistance to TNFα and anti-cancer drug-induced apoptosis [18]. Here, we sought to investigate whether Id1 confers pro-survival properties in NP69 cells, a nasopharyngeal epithelial cell line that is responsive to TGFβ-mediated growth inhibition [19]. Using both cell-cycle and proliferation assays, we found that stable expression of Id1 in NP69 cells enhanced cell proliferation and overcame TGFβ-mediated G1 cell cycle arrest (Figure 3A &3B). Inhibition of TGFβ-mediated growth arrest by LMP1 in B cells and fibroblasts has been reported previously [10,11]. Using an epithelial cell model, we set out to explore whether the resistance to TGFβ afforded by LMP1 was associated with increase expression of Id1. Firstly, NP69-pLNSX and NP69-LMP1 cells were transduced with retroviruses containing either individual shRNAs to Id1 (shId1B or shId1C), or both (shId1B+C). After drug selection, the suppressive function of Id1 shRNAs in stably established cell lines was validated [see Additional file 1].

Using cell cycle analysis, NP69-LMP1 pSuper.retro cells maintained in normal KSF medium were found to contain higher percentage of cells in the S and G2 phases of the cell cycle compared to NP69-pLNSX pSuper.retro control cells, demonstrating that LMP1 promotes cell proliferation (Figure 3C). NP69-LMP1 pSuper.retro cells grown in normal medium (31.1%) or in medium supplemented with TGFβ (30.1%) showed similar percentage of cells in the G1 phase of the cell cycle, while the NP69-pLNSX pSuper.retro control cells treated with TGFβ had higher percentage of cells in the G1 phase (50.1%) compared to untreated cells (38.4%). These findings confirm the ability of LMP1 to protect against TGFβ-mediated G1 cell cycle arrest in this nasopharyngeal epithelial cell line (Figure 3C). The role of Id1 in this response was established as NP69-LMP1 cells expressing shRNAs to Id1 exhibited clear-cut cell cycle arrest, with 48.1% of the cell population in the G1 phase compared to NP69-LMP1 Super.retro, where 30.1% of the cell population was in the G1 phase (Figure 3C). This finding was further supported by MTT cell proliferation assays. As shown in Figure 3D, NP69-LMP1 pSuper.retro cells were relatively refractory to TGFβ-mediated growth inhibition. However, silencing Id1 by shRNA reduced the growth of NP69-LMP1 cells in normal medium (-17.92%) and the growth inhibition was increased further in the presence of TGFβ (-45.65%). Taken together, these data confirm that Id1 plays a significant role in LMP1-mediated cell proliferation and resistance to the growth inhibitory effects of TGFβ.

### Id1 induction by LMP1 confers resistance to TGFβ-mediated transcription

**Figure 4:**
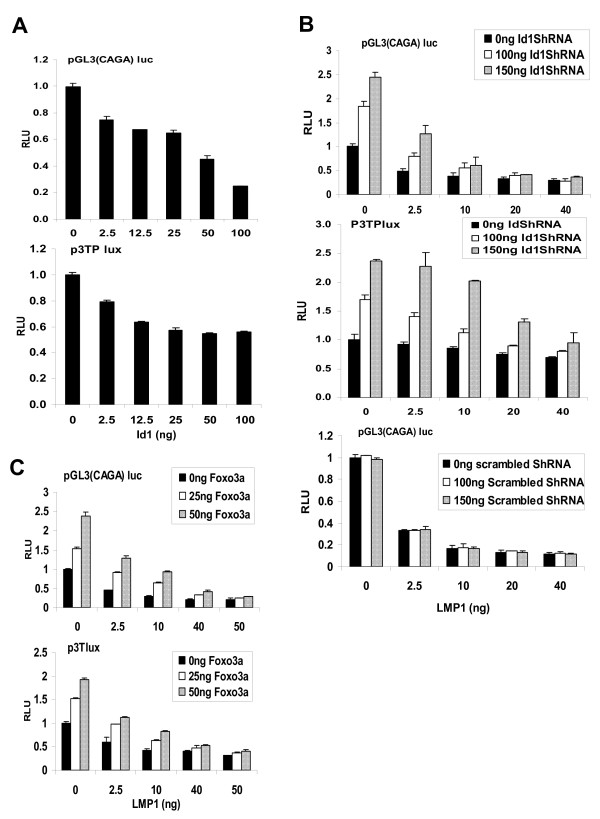
**The involvement of LMP1 induced Id1 in suppressing TGFβ downstream signalling**. (A) HEK-293 cells were transfected with the SMAD-responsive luciferase reporter constructs: pGL3(CAGA) or p3TPlux together with various doses of an Id1 expression vector (pCDNA3-Id1). (B) HEK-293 cells were transfected with pGL3(CAGA) or p3TPlux and various doses of LMP1 expression vector together with Id1 shRNA (shId1B+C) or control scrambled shRNA. (C) HEK-293 cells were transfected with pGL3(CAGA) or p3TPlux and various doses of LMP1 expression vector together with or without a Foxo3a expression vector (pGFP-Foxo3a). Twenty-four hours post-transfection, cells were treated with 10 ng TGFβ in medium with 0.2% FBS for 16 hrs prior to harvesting and luciferase analysis.

To determine whether Id1 confers resistance to TGFβ-mediated cytostasis by inhibiting TGFβ-mediated SMAD transcription, an Id1 expression vector was co-transfected along with the SMAD-responsive reporter construct, pGL3(CAGA) or p3Tplux into HEK-293 cells. Twenty-four hours post-transfection, cells were subjected to TGFβ treatment for 16 hours prior to harvesting for luciferase reporter analysis. As shown in Figure 4A, increased expression of Id1 resulted in a dose-dependent reduction of TGFβ-induced SMAD transcription. This experiment was also performed in HepG2 hepatocellular liver carcinoma cells and NP69 nasopharyngeal epithelial cells, where similar findings were observed [see Additional file 2]. All these results suggest that Id1 is able to inhibit the TGFβ-SMAD-mediated transcription.

LMP1 has previously been reported to inhibit SMAD transcription [12,13]. Here, we reveal that induction of Id1 by LMP1 plays a direct role in this inhibition. As shown in Figure 4B, expression of LMP1 in HEK-293 cells suppressed TGFβ-mediated transcriptional activity of pGL3(CAGA) and p3Tlux reporter constructs. However, addition of Id1 shRNA to silence the expression of Id1 antagonised this suppressive effect, while scrambled shRNA treatment showed no such effect. Similar findings were also observed in HepG2 and NP69 cells [see Additional file 3]. The effects of Id1 silencing are similar to the effects of Foxo3a activation, as Foxo3a has been shown to negatively regulate the expression of Id1 [6]. Exogenous expression of Foxo3a also antagonised the suppressive effect of LMP1 on TGFβ-mediated SMAD transcription (Figure 4C and see Additional file 4). In summary, LMP1 induction of Id1 participates in suppressing the TGFβ-SMAD-mediated transcription.

### LMP1 suppresses the expression of TFGβ-induced p21 and ATF3

**Figure 5:**
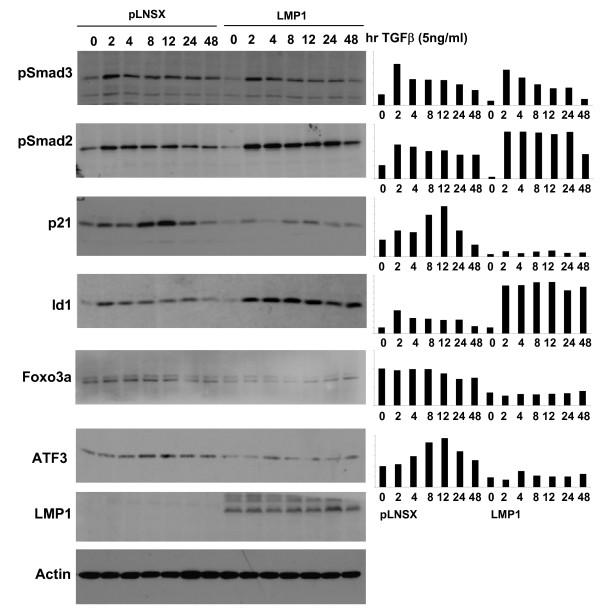
**LMP1 inhibits expression of the TGFβ target genes p21 and ATF3**. NP69-pLNSX and NP69-LMP1 cells were subjected to TGFβ treatment over a course of 48 hr prior to harvesting for immunoblotting. Total cell lysates were subjected to western blot assay. The relative signal intensities of detected proteins are presented in a bar chart.

We have found that LMP1 suppresses TGFβ-mediated SMAD transcription without affecting SMAD phosphorylation. Over a time-course of 48 hours, TGFβ treatment stimulated phosphorylation of the SMAD2 and SMAD3 proteins in both NP69-pLNSX and NP69-LMP1 cells as early as 2 hours after the addition of TGFβ and high levels of phosphorylated SMAD proteins persisted thereafter although gradually declining by 48 hours post-stimulation (Figure 5). Expression of p21 protein, a downstream target of TGFβ who expression is required for TGFβ-mediated cytostasis, gradually increased in NP69-pLNSX control cells 2 hours after TGFβ treatment and reached its peak, with almost a three fold induction, at 12 hours. Its expression then declined to basal levels by 48 hours post-stimulation. In NP69-LMP1 cells, a relatively modest induction of p21 protein was observed upon TGFβ treatment; however, the overall p21 protein level in NP69-LMP1 was significantly lower compared to that in NP69-pLNSX control cells. These findings demonstrate that LMP1's suppressive effect on TGFβ-mediated induction of p21 is independent of SMAD phosphorylation, suggesting that the suppressive effect of LMP1 on SMAD transcriptional activity does not involve formation of activated SMAD complex.

We found that expression of the Id1 protein increased in both NP69-pLNSX and NP69-LMP1 cells 2 hrs after TGFβ addition. Thereafter, high levels of Id1 persisted in NP69-LMP1 cells, while in NP69-pLNSX cells, the levels of Id1 protein gradually decreased reaching basal levels 48 hours post-stimulation. During the time course following TGFβ treatment, the levels of Foxo3a did not change significantly in either NP69-pLNSX or NP69-LMP1 cells although the overall levels of Foxo3a protein were lower in NP69-LMP1 compared to NP69-pLNSX cells. These data show that Id1 is induced in LMP1-expressing cells in response to TGFβ stimulation and that this induction is not likely associated with the expression and/or activity of Foxo3a.

**Figure 6:**
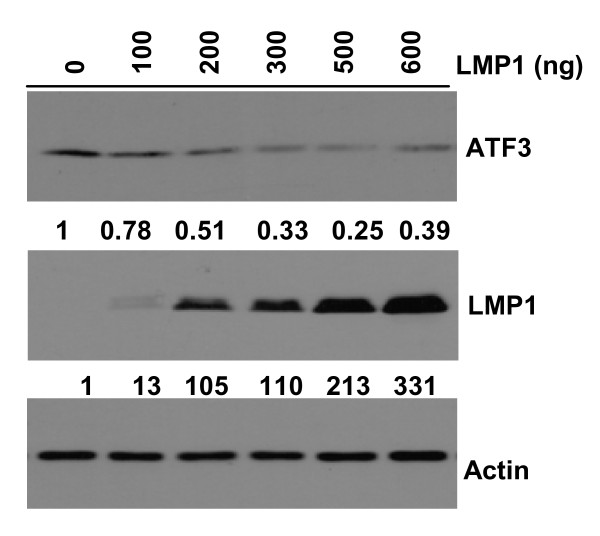
**LMP1 suppresses the expression of ATF3 in a dose dependent manner**. NP69 cells were transfected with various doses of LMP1 expression vector. Forty-eight hours post-transfection, cells were harvested and immunoblotting performed for the ATF3 protein. Relative protein expression was calculated using densitometry with the control set at 1.

Massagué and colleagues have demonstrated that Id1 is transiently induced by TGFβ-activated SMAD3 but long-term TGFβ stimulation results in Id1 transcriptional repression, which is dependent on induction of the ATF3 transcriptional repressor [20]. Here, we found that the basal levels of ATF3 were low in NP69-LMP1 cells relative to NP69-pLNSX cells. After addition of TGFβ, the expression of ATF3 increased in NP69-pLNSX cells at 4 hours and peaked at 12 hours, while in NP69-LMP1 cells, ATF3 protein was slightly increased at 4 hours but was reduced thereafter. This finding suggests that LMP1 inhibition of ATF3 may prolong TGFβ-mediated induction of Id1. The effect of LMP1 on ATF3 suppression was further confirmed in NP69 cells, where transfection of LMP1 suppressed ATF3 protein expression in a dose dependent manner (Figure 6).

### Inactivation of Foxo3a and induction of Id1 in LMP1-expressing NPC tumours

**Figure 7:**
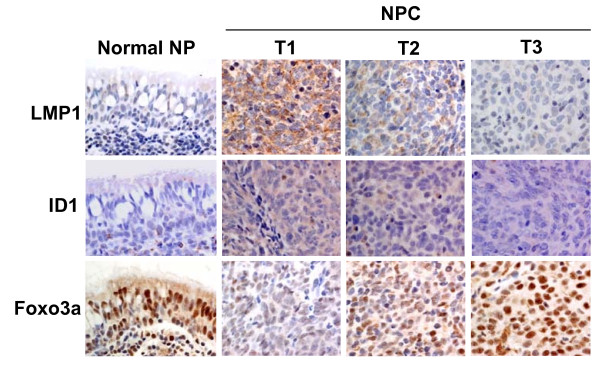
**Immunohistochemical examination of NPC and normal nasopharyngeal epithelium (Normal NP) specimens for LMP1, Id1 and Foxo3a expression**. Intensive nuclear staining for Foxo3a was observed in normal NP epithelium with weak expression of Id1. In NPC tumour T1 which expresses high level of LMP1, strong Id1 expression and weak nuclear staining of Foxo3a was observed. In NPC tumour T3 which shows weak LMP1 expression, strong nuclear staining of Foxo3a and weak Id1 detection was observed.

In an examination of primary NPC tumours (T1, T2 and T3), which displayed strong, moderate, and weak expression of LMP1 respectively, we observed a positive correlation between expression of LMP1 and that of Id1, whereas expression of Foxo3a was inversely correlated with LMP1 expression. For example, tumour T1 shows strong staining for both LMP1 and Id1, but weak Foxo3a nuclear staining. In contrast, tumour T3 showed strong nuclear staining of Foxo3a but weak detection of LMP1 and Id1 proteins. While in the normal nasopharyngeal epithelium (NP) which is LMP1 negative, we found weak Id1 expression but strong nuclear Foxo3a staining (Figure 7). These data suggest that LMP1 is involved in suppressing Foxo3a activity and increasing Id1 expression during NPC progression.

## Discussion

**Figure 8:**
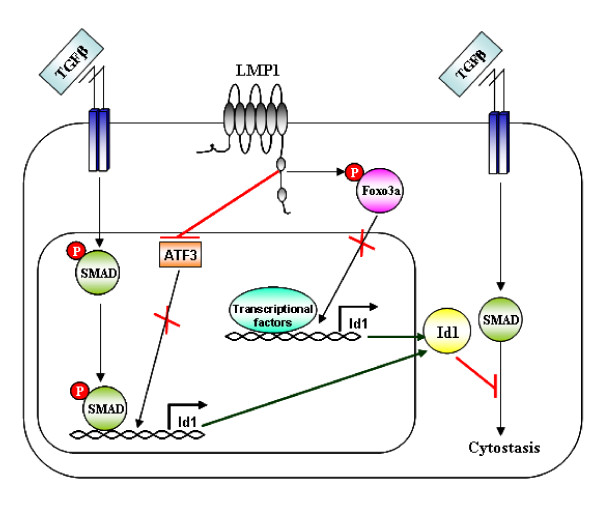
**Proposed model describing the mechanism by which LMP1 suppresses TGFβ-mediated cytostasis**.

The EBV-encoded LMP1 protein is oncogenic and exerts various transforming effects in both lymphoid and epithelial cells. LMP1-mediated cellular transformation confers resistance to TGFβ-mediated growth arrest and modulates SMAD transcriptional activity [10-13]. LMP1 also increases expression of Id1, a HLH protein whose deregulation plays a role in carcinogenesis and suppresses the activity of Foxo3a transcription factor which is responsible for controlling cell integrity and homeostasis [14,16]. In this study, we demonstrate that phosphorylation and inactivation of the Foxo3a transcription factor by LMP1 leads to Id1 upregulation. Our finding that LMP1 expression in primary NPC tumours correlates with reduction of activated Foxo3a in the nucleus and increased expression of Id1 corroborates findings obtained from our *in vitro *studies. Also, we have found that the LMP1 induction of Id1 contributes to resistance to TGFβ-mediated cytostasis and modulate TGFβ-SMAD-mediated transcription (Figure 8). Although LMP1 did not interfere with TGFβ-induced SMAD phosphorylation, it impaired SMAD-dependent transcription and suppressed induction of the TGFβ-induced growth inhibitory protein p21. TGFβ is known to negatively regulate Id1 transcription through a mechanism involving SMAD3 activation and induction of the transcription repressor, ATF3 [8,9]. Here, we report that LMP1 inhibits basal and TGFβ-induced ATF3 expression. Suppression of ATF3 by LMP1 abolishes the repressive effect of TGFβ to Id1 expression (Figure 8). Our current findings provide new insights into the mechanism by which LMP1 counteracts the cytostatic action of TGFβ and underscore the importance of Id1 in LMP1-mediated cell transformation.

Id1 proteins are important regulators of cellular differentiation and cell cycle progression. Over-expression of Id1 has been widely observed in human cancers where it may play a critical role in tumourigenesis and cancer progression [7]. Previous studies have demonstrated upregulation of Id1 by LMP1 in culture epithelial cell lines [16,17]. Here, an examination of NPC primary tumours revealed a positive correlation between LMP1 and Id1 expression in NPC cells. In a recent report, Raab-Traub and colleagues have established that silencing Id1 affects the growth of LMP1-transformed and parental Rat-1 fibroblasts [15]. However, the precise contribution of Id1 to LMP1-mediated transformation is not clear. In the present study, we reveal that Id1 increased cell proliferation and conferred resistance to TGFβ-mediated cell cycle arrest in nasopharyngeal epithelial cells. Silencing Id1 by shRNA abolished LMP1-mediated cellular growth advantage and TGFβ resistance. These findings identify the important contribution of Id1 in cell growth control and resistance to TGFβ, and suggest that the induction of Id1 by LMP1 plays a key role in epithelial cell growth transformation.

TGFβ-induced cytostasis is mediated at least in part by SMAD-dependent transcriptional regulation. Activated SMAD complexes cooperate with various transcription factors to regulate the expression of TGFβ target genes involved in growth inhibition and apoptosis [8,9]. In this study, we found that silencing Id1 diminishes the ability of LMP1 to inhibit TGFβ-mediated SMAD transcriptional activity, indicating that Id1 plays an essential role in this inhibition. Id1 proteins contain a HLH domain that allows them to negatively regulate bHLH transcription factor family members. Although the mechanism of Id1 in suppressing SMAD transcriptional activity is not clear, it is possible that Id1 interferes certain bHLH transcription factors involved in SMAD-mediated transcription. In fact, a similar scenario has recently been reported showing that an Id-like HLH protein, human homologue of Maid (HHM) suppresses TGFβ-mediated cytostasis and TGFβ-induced expression of PAI-1, PDGF-β and p21 by inhibiting the binding of Olig to Smad2/3 [21]. Olig is a bHLH transcription factor involved in TGFβ-SMAD mediated transcription.

The cyclin dependent kinase inhibitor p21 protein is one of the major TGFβ-activated targets responsible for cell growth inhibition. Our previous report showed that p21 protein expression is suppressed in LMP1-expressing nasopharyngeal epithelial cells [22]. Other workers have also found that LMP1 inhibits both basal and SMAD-induced activity of the p21 promoter [12]. Here, we further confirm that LMP1 suppresses the expression of TGFβ-induced p21 protein. Although the mechanism of p21 suppression by LMP1 is not clear, it may be associated with Id1 induction as several reports indicate that Id1 is able to restrain p21 [18,23]. The impact of Id1 on SMAD mediated p21 expression is clearly an area worthy of further investigation.

TGFβ-activated SMAD proteins interact with a large number of DNA binding cofactors, coactivators, and corepressors, to target different genes with high affinity and specificity. The outcome of TGFβ-induced effects is determined by the availability of activated SMAD proteins as well as DNA binding transcriptional factors. Previous reports have found that LMP1 does not affect degradation and nuclear localisation of the SMAD protein [12]. LMP1 also fails to affect the formation of SMAD heteromers as well as DNA binding activity of SMAD protein [12,13]. Therefore, it is not surprising we find that the inhibitory effect of LMP1 on transcriptional activity is independent of SMAD phosphorylation. This also suggest that the suppressive effect of LMP1 on SMAD transcriptional function is not due to inhibition of TGFβ-activated SMAD signalling and may be owing to repression of the transcriptional cofactors involved in SMAD-mediated transcription. Here, we show that LMP1 modulates expression of transcription repressor ATF3 that cooperates with SMADs to regulate gene transcription.

During TGFβ-mediated cytostasis, TGFβ-mediated SMAD signalling also results in the transcriptional repression of the growth promoting genes inducing c-Myc, Id1, Id2 and Id3[8,9]. In response to TGFβ stimulation, SMAD signalling rapidly induces ATF3 expression. ATF3 then associates with SMAD complex to target Id1 for transcriptional repression. Dominant-negative ATF3, which is able to compete with endogenous ATF3 for binding to SMAD3 and the Id1 promoter has been found to abolish Id1 transcriptional repression by TGFβ [20]. This indicates that ATF3 is necessary for TGFβ-mediated Id1 repression. In the absence of ATF3, TGFβ-activated SMAD3 binds to Id1 promoter directly, leading to Id1 upregulation [20]. In this study, we found that ATF3 protein expression is suppressed by LMP1 resulting in prolonged induction of Id1 by TGFβ. Upregulation of Id1 by TGFβ has been reported in various cell types including fibroblasts, endothelial cells, renal epithelial cells, and hepatic stellate cells [24-27]. The possible association with the absence of ATF3 in these cell types awaits further investigation. In addition to its role in TGFβ-mediated Id1 repression, ATF3 also functions to suppress tumour growth. Previous studies indicate that overexpression of ATF3 results in increased apoptosis of prostate cancer cells, reduced tumour size of colorectal xenografts in nude mice, and increased apoptosis and reduced metastatic potential of ovarian cancer cells [28-30]. The mechanism of ATF3 suppression mediated by LMP1 will be examined further.

## Conclusions

Id1 is a critical downstream target of LMP1 and likely plays an important role in mediating growth transformation. Here we show that LMP1 inactivates the function of Foxo3a leading to the induction of Id1. LMP1 also inhibits the expression of the ATF3 transcription repressor, preventing the suppression of Id1 by TGFβ-mediated SMAD signalling. The induction of Id1 by LMP1 confers a growth advantage to LMP1-expressing cells, by rendering cells refractory to the cytostatic effects of TGFβ. Our findings provide a possible therapeutic strategy whereby inactivation of Id1 may lead to sensitisation of LMP1-positive NPC cells to chemotherapeutic drug induced apoptosis.

## Methods

### Cell lines, chemicals and transfection

HEK-293 and HepG2 cells were grown in DMEM medium (Sigma) supplemented with 10%v/v FBS, and antibiotics. NP69 nasopharyngeal epithelial cells were maintained in Keratinocyte-Serum free (KSF) medium (Invitrogen). Recombinant human TGF-β1 (PeproTech) and MG132 (Sigma) were used for treatment of cells when specified. Plasmid transfections were performed using either Fugene HD (Roche) or TurboFect™ *in vitro *Transfection Reagent (Fermentas) according to manufacturers' instructions.

### DNA constructs

The Id1 (-1695) and (-353) promoter luciferase reporters were generated by PCR and cloned into the pGL3 basic vector (Promega). The Id1 shRNA B & C expression vectors were generated by inserting a fragment of synthesised Oligo (Sigma) with the sequence of Id1 coding region into pSUPER.retro.puro vector (oligoengine). The sequence of Id1 shRNA B is GATCCCCGCGCGCTGAAGGCCGGCAATTCAAGAGATTGCCGGCCTTCAGCGCGCTTTTTA and Id1 shRNA C is GATCCCCGGTGCGCTGTCTGTCTGAGTTCAAGAGACTCAGACAGACAGCGCACCTTTTTA. pECE-HA-Foxo3a was kindly provided by M. Deckert [31], pGL2-Bim vector is gift of P.J. Coffer [32], GFP-Foxo3a is a gift from MC Hung [33]. The pGL2-p27kip promoter construct was provided by T Sakai [34]. P3Tplux was provided by J Massagué Laboratory, Memorial Sloan-Kettering Cancer Center, New York, USA and pGL3(CAGA) was kind gift from CS Hill (The Cancer Research UK London Research Institute, London, UK).

### Western blotting analysis

The detailed procedures of Western blotting have been described previously [35]. Briefly, cells were lysed in RIPA buffer. Total cell lysates (5-25 μg of protein) were separated by 10% SDS-PAGE and then electrophoretically transferred to nitrocellulose membrane prior to immunoblotting. Antibodies specific for Phospho-IκBα (Ser32), Phospho-Erk1/2 (Ser217/221), phospho-Akt (Ser473), phospho-Foxo3a (Ser253), Foxo3a and p27 were purchased from Cell Signalling, USA. Antibody specific for ATF3 was from Abcam. Antibodies to SP-1 and α-tubulin were purchased from Santa Cruz, USA. Antibodies to LMP1 were purchased from Dako and β-Actin from Sigma, UK.

### Immunohistochemistry

The expression of LMP1 and Foxo3a in paraffin-embedded NPC specimens was examined by immunohistochemistry as described previously [36]. Primary antibodies used in this study were anti-LMP1 mouse monoclonal antibody (DAKO) and anti-Foxo3a rabbit polyclonal antibody (Cell Signalling).

### Luciferase reporter assay

1×10^5 ^cells grown in 24-well plates were co-transfected with 40 ng of luciferase reporter constructs together with different amounts of expression vectors as indicated in the text. RSV-β-Gal vector (50 ng) was transfected as an internal control to normalise for transfection efficiency. Two days post-transfection, cells were lysed in reporter lysis buffer (Promega) and then assayed for luciferase and β-gal activities. For detection of Id promoter activity, transfected cells were cultured in serum free medium for 6 hrs before harvesting. For detection of TGFβ responsive promoter activity of pGL3(CAGA) and p3TLux constructs, cells were cultured in medium containing 0.2% FBS and 5 ng/ml TGFβ for 16 hrs prior to harvesting.

### Cell Cycle Analysis

Cells (5 × 10^5^) were fixed in ice cold 70% ethanol for 1 hr. Prior to analysis, fixed cells were washed with PBS, treated with RNase (1 μg/ml) and stained with propidium iodide (50 μg/ml) for 30 min at 37°C. Cell cycle analysis was carried out on a XL-MCL flow cytometer (Beckman-Coulter) and data analyzed using the MultiCycle AV DNA Analysis software (Phoenix Flow Systems).

### MTT assay

For MTT assay, cells (5×10^3 ^per well) were seeded into 96-well plates. One day after cell seeding. TGFβ1 (10ng/ml) was added. MTT assay was analyzed each 24 hrs by adding MTT solution (5 mg/ml; 10 μl/well) and cells were incubated at 37°C for 5 hrs. The culture media were aspirated and DMSO (200 μl/well) was added to dissolve the formazan crystals. The absorbance was measured at a wavelength of 570 nm. Each time point was performed in triplicate. Results are presented as relative growth rate by dividing the absorbance value of the cells at indicated time points by the absorbance value of the cells one day after cell plating. Each data point is represented by the mean and SD.

## Competing interests

The authors declare that they have no competing interests.

## Authors' contributions

AKFL conceived the study, participated in its design, performed most of the experimental work and drafted the manuscript. CWD participated in the experimental design, interpretation of the results and helped to draft the manuscript. KWL performed immunohistochemical staining and data interpretation and helped to draft the manuscript. CWD and KWL equally contributed to the present work. YY generated part of expression vectors and assisted in their validation. LSY participated and coordinated the study, compiled and finalized the manuscript. All authors read and approved the final manuscript.

## Supplementary Material

Additional file 1 **Validation of Id1 expression in Id1 shRNA expressing NP69 cells**. NP69-pLNSX and NP69-LMP1 cells were transduced with pSuper.retro control or pSuper.retro-Id1 shRNA B (shId1B), pSuper.retro-Id1 shRNA C (shId1C) or two shRNA-Id1 constructs (shId1B+C). The sequences of Id shRNA B and C are described in Material and Methods. After Puromycin drug selection, Id1 shRNA expressing cells were validated for Id1 expression by western blotting. Click here for file

Additional file 2 **Id1 suppresses TGFβ-mediated SMAD transcriptional activity**. NP69 or HepG2 cells were transfected with SMAD-responsive luciferase reporter construct pGL3(CAGA) together with various doses of an Id1 expression vector. Twenty-four hours post-transfection, cells were treated with 10 ng TGFβ in medium supplemented with 0.2% FBS for 16 hrs prior to harvesting for luciferase analysis. Click here for file

Additional file 3 **LMP1 induction of Id1 suppresses TGFβ-mediated SMAD transcriptional activity**. NP69 or HepG2 cells were transfected with pGL3(CAGA) or p3TPlux and various doses of LMP1 expression vector together with Id1 shRNA (shId1B+C). Twenty-four hours post-transfection, cells were treated with 10 ng TGFβ in medium with 0.2% FBS for 16 hrs prior to harvesting for luciferase analysis. Click here for file

Additional file 4 **LMP1 induction of Id1 suppresses TGFβ-mediated SMAD transcriptional activity**. NP69 or HepG2 cells were transfected with pGL3(CAGA) and various doses of LMP1 expression vector together with a Foxo3a expression vector. Twenty-four hours post-transfection, cells were treated with 10 ng TGFβ in medium supplemented with 0.2% FBS for 16 hrs prior to harvesting for luciferase analysis. Click here for file
