# Supplementary figures and images for: Upregulation of Id1 by Epstein-Barr Virus-encoded LMP1 confers resistance to TGFβ-mediated growth inhibition
Source: Mol Cancer. 2010 Jun 18;9:155. doi: 10.1186/1476-4598-9-155 (PMC2908095; doi:10.1186/1476-4598-9-155)

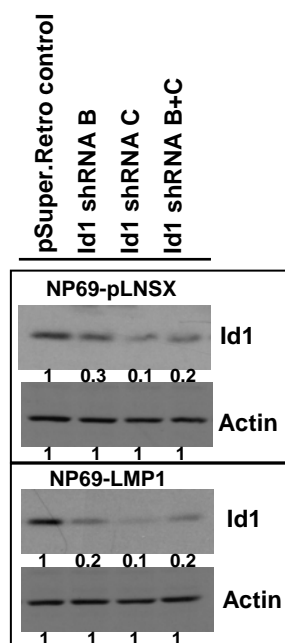

Supplement: Additional file 1 — Validation of Id1 expression in Id1 shRNA expressing NP69 cells. NP69-pLNSX and NP69-LMP1 cells were transduced with pSuper.retro control or pSuper.retro-Id1 shRNA B (shId1B), pSuper.retro-Id1 shRNA C (shId1C) or two shRNA-Id1 constructs (shId1B+C). The sequences of Id shRNA B and C are described in Material and Methods. After Puromycin drug selection, Id1 shRNA expressing cells were validated for Id1 expression by western blotting. [file 1476-4598-9-155-S1.PDF]

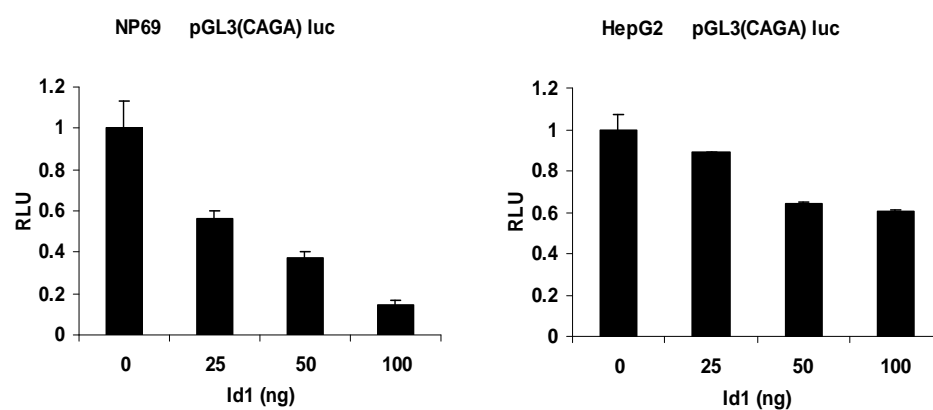

Supplement: Additional file 2 — Id1 suppresses TGFβ-mediated SMAD transcriptional activity. NP69 or HepG2 cells were transfected with SMAD-responsive luciferase reporter construct pGL3(CAGA) together with various doses of an Id1 expression vector. Twenty-four hours post-transfection, cells were treated with 10 ng TGFβ in medium supplemented with 0.2% FBS for 16 hrs prior to harvesting for luciferase analysis. [file 1476-4598-9-155-S2.PDF]

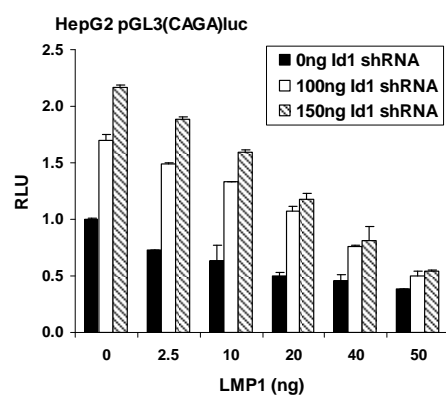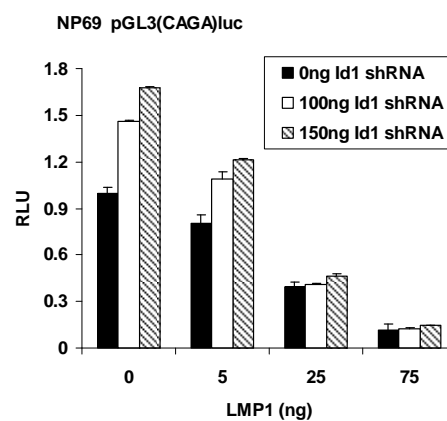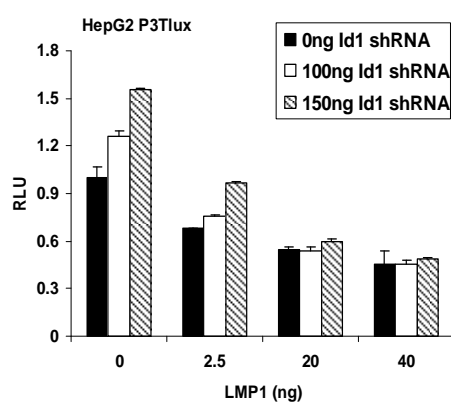

Supplement: Additional file 3 — LMP1 induction of Id1 suppresses TGFβ-mediated SMAD transcriptional activity. NP69 or HepG2 cells were transfected with pGL3(CAGA) or p3TPlux and various doses of LMP1 expression vector together with Id1 shRNA (shId1B+C). Twenty-four hours post-transfection, cells were treated with 10 ng TGFβ in medium with 0.2% FBS for 16 hrs prior to harvesting for luciferase analysis. [file 1476-4598-9-155-S3.PDF]

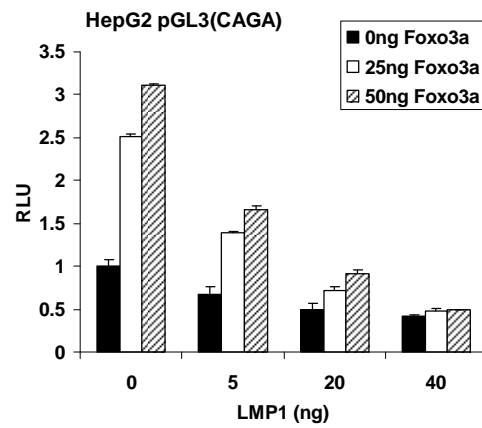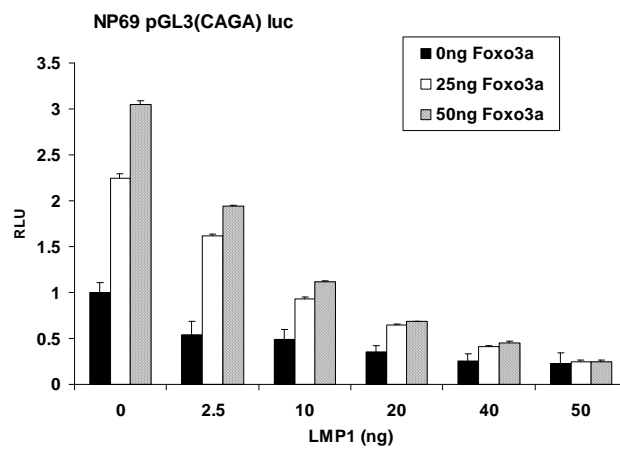

Supplement: Additional file 4 — LMP1 induction of Id1 suppresses TGFβ-mediated SMAD transcriptional activity. NP69 or HepG2 cells were transfected with pGL3(CAGA) and various doses of LMP1 expression vector together with a Foxo3a expression vector. Twenty-four hours post-transfection, cells were treated with 10 ng TGFβ in medium supplemented with 0.2% FBS for 16 hrs prior to harvesting for luciferase analysis. [file 1476-4598-9-155-S4.PDF]
